# A Theoretically Based Model of Rat Personality with Implications for Welfare

**DOI:** 10.1371/journal.pone.0095135

**Published:** 2014-04-22

**Authors:** Becca Franks, E. Tory Higgins, Frances A. Champagne

**Affiliations:** Department of Psychology, Columbia University, New York, New York, United States of America; University of Houston, United States of America

## Abstract

As animal personality research becomes more central to the study of animal behavior, there is increasing need for theoretical frameworks addressing its causes and consequences. We propose that regulatory focus theory (RFT) could serve as one such framework while also providing insights into how animal personality relates to welfare. RFT distinguishes between two types of approach motivation: promotion, the motivation to approach gains, and prevention, the motivation to approach or maintain safety. Decades of research have established the utility of RFT as a model of human behavior and recent evidence from zoo-housed primates and laboratory rats has suggested that it may be applicable to nonhuman animal behavior as well. Building on these initial studies, we collected data on 60 rats, *Rattus norvegicus*, navigating an automated maze that allowed individuals to maintain darkness (indicative of prevention/safety-approach motivation) and/or activate food rewards (indicative of promotion/gain-approach motivation). As predicted, both behaviors showed stable individual differences (Ps <0.01) and were inversely associated with physiological signs of chronic stress, possibly indicating poor welfare (Ps <0.05). Subsequently, half the rats were exposed to a manageable threat (noxious novel object) in the homecage. Re-testing in the maze revealed that threat exposure increased darkness time achieved (P<0.05), suggesting a mechanism by which prevention motivation may be enhanced. These results point toward the potential utility of RFT as a model for animal behavior and welfare.

## Introduction

Personality research in nonhuman animals, or more generally, the study of individual differences in animal behavior, is now established as an important line of inquiry in a broad range of academic fields including behavioral ecology [1], [2], behavioral biology [3], [4], psychology [5], [6], and animal welfare science [7]. With this increasing interest, there is increasing need for theoretical frameworks addressing the causes and consequences of animal personality [8]–[10]. Several definitions and theoretical conceptions of personality exist across the various fields of research [5], [11], [12]. Here we use personality to describe cases in which individuals are consistent in their behavior over time–in other words, cases in which the individual is a significant source of variability in behavior [13]. We propose that along with other theories from behavioral ecology and biology, regulatory focus theory (RFT) [14], a theory that describes the dynamics of approach motivation, may contribute to a deeper understanding of personality and welfare.

RFT describes two distinct approach motivations: *prevention*, the motivation to secure and maintain safety and *promotion*, the motivation to acquire and maximize gains [14]. Such a framework provides insights that would not be possible with a hedonic model of behavior (approach pleasure, avoid pain). For example, in typical or neutral situations, RFT states that having a promotion (gain) motivation will increase the probability of approaching risky outcomes or engaging in risky behavior [15]. However, a strong prevention motivation can also lead to risky behavior. In studies where human participants were in a state of monetary loss in a stock investment scenario, having a prevention (safety) motivation *increased* the probability of making (approaching) risky gambles when those risky gambles were necessary to restore a safe, non-loss state [16]. As such, promotion and prevention motivations also do not map neatly onto the bold-shy continuum, a widely studied model of animal personality that is operationalized as the propensity to engage in risky behavior [17], [18]. Moreover, unlike the bold-shy continuum, human research has shown that being effective (successful) in promotion related goals and being effective in prevention related goals are positively associated such that individuals who are effective/ineffective in one also tend to be effective/ineffective in the other [15], [19].

Recent studies have indicated that nonhuman animals may also have promotion and prevention ‘personalities’ [20], [21]. For example, across multiple tests in a large, square arena, rats spent a consistent amount of time in a location associated with gains (palatable food rewards) and a consistent amount of time in a location associated with safety (darkness for nocturnal animals), suggesting individual differences in promotion (gain) and prevention (safety) motivations [21]. Furthermore, paralleling the human research, individual differences in safety/prevention motivation predicted risky behavior in a separate context. Rats with the highest darkness times in the test arena (reflecting a prevention motivation) spent the longest duration of time in close proximity to a threatening noxious novel object (NNO), a risky behavior that was necessary to contain the threat by burying it, a rat’s natural defensive behavior [4], [22]. Promotion motivation (gain pursuit in the test arena) was unrelated to the duration of time spent near the NNO.

Previous research on individual differences in rats has described two important dimensions that bear some similarity to promotion and prevention motivations respectively: novelty-seeking and harm-avoidance [23], [24]. Novelty-seeking describes a consistent tendency to thoroughly explore a relatively novel environment. Harm-avoidance, on the other hand, describes a consistent tendency to minimize exposure to threatening environments, such as brightly lit, elevated areas [24]. Though there is some theoretical overlap between these dimensions and RFT, important differences exist as well. For example, an individual with a promotion orientation will not seek out novelty indiscriminately, but rather only when seeking novelty has a good chance of leading to a gain [14]. This prediction was borne out in previous RFT primate research, which found that promotion was associated with a fast approach to novelty, but only when it was likely to be associated with a gain [20]. Similarly, an individual with a prevention orientation will not avoid harm indiscriminately, but rather only when avoiding harm has the best chance of leading to safety [14]. As described above, previous research in rats, paralleling that in humans [16], found that prevention was associated with longer durations spent with the NNO [21]. In other words, the prevention individuals were the *least* avoidant animals in this test. Finally, as a theory regarding motivation, RFT has the potential to integrate well with welfare theory and research, which also relies on motivational models of behavior [25]–[27]. Thus, RFT may be able to predict unique patterns of behavior in nonhuman animals and, given its motivational nature and its association with well-being in humans [28], may also provide insights into animal welfare as well.

The term “welfare,” like the term “personality,” has been subject to a variety of definitions [29], [30]. Along with many welfare scientists [25], [26], we adopt a motivational perspective on welfare such that good welfare involves a state in which an individual’s strong motivational needs are capable of being met [31]. Research in humans has shown that an individual’s history of promotion and prevention success is associated with greater quality of life (human welfare) [28]. Furthermore, similar research has shown that a history of promotion success leads people to adopt more effective (successful) promotion strategies in the future and that the complimentary pattern exists for the prevention system as well [15]. As many models of animal welfare propose a fundamental link between welfare and an individual’s success at achieving desired outcomes [25], [27], [32], we explored the link between welfare and individual differences in promotion and prevention successes, expecting to find a positive association between welfare and individual differences in promotion success *and* prevention success [31].

Extending the preliminary nonhuman RFT research, we also sought to explore how these individual differences in motivation are sustained. Within behavioral ecology, positive-feedback-loops have been hypothesized to play a critical role in the development and perpetuation of personality [33], [34]. This model of personality posits that stable individual differences can arise from initial state differences if the initial state leads an individual to experience environmental conditions that, in turn, sustain or amplify the state: a positive-feedback-loop. Previous work in rats suggested that prevention motivation led individuals to spend more time with threatening objects [21], the first path of a potential positive-feedback mechanism. The loop would be completed if threatening objects sustain or amplify prevention motivation. The existence of this second path is currently unknown in rats, though consistent with RFT and human research [14]. Thus, in the present research we also experimentally manipulated exposure to NNOs and measured subsequent prevention motivation.

## Methods

### Animals and Husbandry

In these studies, we worked with Long-Evans female rats (N = 60) bred and housed in the animal facility in the Department of Psychology at Columbia University. From the time of weaning (postnatal day 21), rats were group-housed (4/cage) in large cages (38×20×61 cm) with pine shaving bedding and maintained at a constant temperature and humidity with a 12L:12D light schedule (lights on 9∶00). In addition to periodic food enrichment (3–4 times per week of various cereals, fruits, vegetables, nuts, etc.), rat chow and water were available continuously. Each cage contained a large opaque plastic insert that provided shelter and environmental complexity. After completing the tests involved in this study, rats were maintained in these housing conditions for future behavioral studies. All procedures were performed in accordance with guidelines of the NIH regarding the Guide for the Care and Use of Laboratory Animals and with the approval of the Institutional Animal Care and Use Committee (IACUC) at Columbia University.

### Experimental Procedures and Apparatus

**Figure 1:**
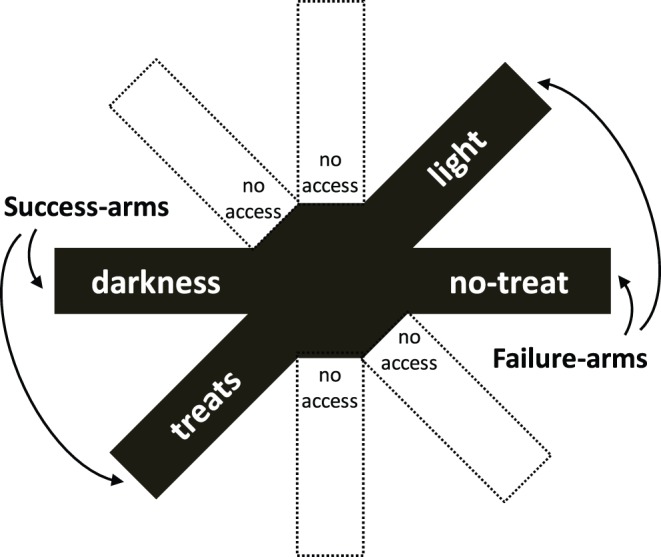
The automated-maze. In this study, four arms of an 8-arm radial arm maze were accessible. Reaching the end of two arms activated successful outcomes: treats (promotion success, i.e., gains) and darkness (prevention success, i.e., safety). Reaching the end of the other two arms activated failure outcomes: no-treats (promotion failure) and lights-on (prevention failure).

Habituation and testing began at postnatal day 60. All procedures were conducted during the light cycle, between 10∶00 and 19∶00. Rats were tested in a radial-arm maze, which contained eight arms projecting from a central hub (ScientificDesign). For this experiment, four arms were blocked from entry (Figure 1). Each of the remaining four arms was accessible and contained contingencies that a computer with AnyMaze software automatically activated when the animal reached the end of the arm. AnyMaze tracked the movement of the rat in real-time via a video camera mounted above the maze. The two RFT success-arms were adjacent, each containing an approach-type goal. Reaching the end of one arm turned off the overhead light for 30 seconds (dark-arm; safety success), after which, the overhead light turned back on automatically. Reaching the end of the other arm released a highly palatable food reward (treat-arm; gain success). The two RFT failure-arms were adjacent and directly opposite the success-arms: reaching the end of one arm turned on the overhead light (light-arm; safety failure) and reaching the end of the other arm activated the food dispenser mechanism without actually dispensing a treat (nontreat-arm; gain failure). Thus, the maze was designed to be a primarily rewarding space with mild and easily avoidable negative outcomes and opportunities to achieve positive outcomes (safety and gains).

### Testing Phase 1: Individual Differences

In Phase 1, we sought to use the automated maze to identify individual differences in regulatory focus success–promotion (treats) and prevention (darkness)–and explore how these individual differences may relate to individual differences in welfare. To minimize the stress of being exposed to a novel environment, before testing began, all four cage-mates were allowed to freely explore (habituate to) the maze together for four minutes with the light off. A week later, testing commenced with each individual tested separately. Each test began with the lights on and lasted ten minutes. These tests were repeated four times for each rat over the course of two weeks. At the end of each individual’s test, fecal boli and uneaten treats were counted and the maze was cleaned with a 70% ethanol solution. The AnyMaze software automatically recorded the amount of time that the light was off (darkness time, prevention success), the number of treat activations (treats, promotion success), and the amount of time the animals spent in each section of the maze–of particular interest, the end of dark-arm, end of treat-arm, end of nontreat-arm, end of light-arm. From the first day of individual testing and continuing throughout the experiment, all animals readily explored the maze, suggesting that being tested individually in the maze induced little stress. The data from days 2–4 were used in the analyses, giving the animal one day to habituate to the apparatus further and experience the success and failure contingencies. With these three tests collected over two weeks, we could examine stability in promotion and prevention successes and relate these individual differences to welfare.

We used boli as an index of stress and, potentially, poor welfare. Previous research has shown that rodents produce more fecal boli in threatening or high-stress situations, suggesting that elevated boli production is indicative of acute states of fear or anxiety [35]–[38]. To validate this measure further, we recorded the environmental conditions prior to testing. One cage was erroneously not tested on the final test day, thus the total number of tests was only 176, not 180. Of the 176 tests, 22 tests were classified as being preceded by a negative disturbance (e.g., a flooded home-cage or water bottle changes outside normal husbandry hours) that would increase stress, allowing us to analyze these events as ‘natural experiments’ of the effect of environmental stress on fecal boli production. To use boli as potential measure of welfare, we reasoned that if present, stably high (vs. stably low) boli production over time would be indicative of an individual experiencing chronic fear or anxiety and thus poor welfare.

### Testing Phase 2: Effect of a Noxious Novel Object (NNO)

In Phase 2, we tested whether exposure to a manageable threat, a NNO, served as a situational induction of prevention (safety) motivation. Two months after Phase 1, approximately half of the rats (N = 32) were given NNOs twice a week in their homecage for three weeks. Rats in the other cages (N = 28) received no novel objects. The NNO was a metallic teabag anchored to the front of the cage and filled with a paper towel soaked in either bleach or household cleaner. Fifteen minutes after placing the novel object in the cage, the cage was scanned for signs of burying behavior, a defensive response in rats [4], [22]. We also scanned the rats’ behavior to ensure that the NNOs were not causing undue, persistent fear. All cages showed signs of burying, but not excessive stress: we never observed acute fear behaviors such as immobilization and behavior typically returned to normal (sleeping, eating, grooming, etc.) within 15 minutes. Thus, we can surmise that the NNOs were moderate, but manageable threats–the least stressful stimulus we could provide while still achieving the desired response. One week after NNO treatments, all animals were tested twice in the automated maze with the same four arms and contingencies described in Phase 1. Testing sessions in Phase 2 lasted for four minutes. During the testing weeks, no novel objects were placed in the cages.

This experimental paradigm also gave us the opportunity to examine the relationship between prevention plasticity and welfare. We expected that rats with high or plastic (flexible and responsive) prevention motivation would show signs of coping better with the NNOs than rigidly low prevention animals (rats with a low prevention score that remained low).

### Statistical Analysis

Multilevel (mixed or random effects) models were used in Stata v12.2 to examine the repeated observations of each animal [39], [40]. As an outcome variable (dependent variable), we investigated time in dark-arm, treat-arm, light-arm, nontreat-arm, center, darkness achieved, treats activated, and fecal boli. All models included a random intercept, which controls for repeated observations and can test for individual stability in response level [40]. For count data (boli and treats), a generalized multilevel model with a log-link and Poisson error distribution was used. In all multilevel models, experimental day was centered such that the intercept of the model was the predicted level of behavior on the second day examined; the first test day in the maze was coded as −1, the second as 0, and the third as +1. Thus, all models in Phase 1 included, at a minimum, test day as a fixed-effect and individual as random intercept. Additional fixed effects were added to this base model to test specific predictions as indicated below. As generalized multilevel models assume an asymptotic sampling distribution and as our sample size was sufficiently large (N = 60), all tests of fixed effects are reported with a *z*-statistic [40], [41].

The multilevel nature of the data also allowed for the investigation of between-subject (individual) vs. within-subject (situational) effects [41], [42]. By this methodology, two orthogonal variables–(i) each individual’s average response and (ii) the daily deviation from that average response–are calculated and added to the fixed-effects portion of the model (always including a random intercept by individual). This parameter specification mitigates the ecological fallacy (a.k.a. Simpson’s paradox), which is the hazard of conflating effects at one level of analysis (e.g. individual organism) with effects at another level of analysis (e.g. situational variability). Thus, this modeling approach can begin to indicate structural relationships between two variables. Specifically, we were interested in determining the level at which promotion and prevention success relates to welfare. We predicted an individual’s history of promotion and prevention success would lead to more effective behaviors in the maze *and* better welfare, producing an individual-level association between welfare and promotion success (treat activations) and prevention success (darkness time).

To look for stability across the two phases, Pearson correlation coefficients were calculated for average darkness and average treats. To determine the effect of the NNO, we conducted *t*-tests comparing groups on prevention and promotion behavior after exposure to the NNO.

## Results

### Testing Phase 1: Individual Differences

#### Characterizing promotion and prevention in rats

**Figure 2:**
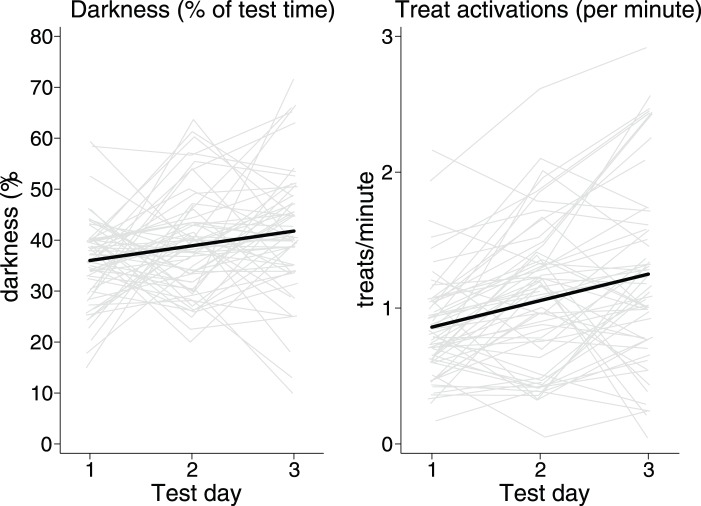
Individual differences in prevention (safety) and promotion (gains) success (black lines are population trends; grey lines are individual trajectories). Over the course of 3 test days, rats achieved progressively more darkness and treats (*P*<0.01 and *P*<0.001, respectively). They also showed significant individual differences in their mean responses of both behaviors (*P* = 0.001 and *P*<0.001, respectively).

Over the course of the three test days, rats spent progressively more time in the dark-arm and treat-arm (*z* = 2.78, *P* = 0.005; *z* = 4.29, *P*<0.0001, respectively) and progressively less time in the light-arm and nontreat-arm (*z* = 5.85, *P*<0.0001; *z* = 5.87, *P*<0.0001, respectively). As would be expected, the increased time in the success-arms and decreased time in the failure-arms resulted in more positive outcomes across the test days: the rats achieved significantly more darkness time (*z* = 3.29, *P* = 0.001; Figure 2) and more treats (*z* = 6.26, *P*<0.0001; Figure 2). Furthermore, we found that rats spent significantly less time in the center of the maze across the three test days (*z* = 4.40, P<0.0001). These results suggest that the success-arms were positively reinforcing, approach-type outcomes, and therefore potentially good measures of a prevention motivation (darkness time) and promotion motivation (treats). Finally, beyond this population-level pattern of reinforcement, random effects indicated that some rats pursued these outcomes more than others: we found strong individual differences in prevention success and promotion success (χ^2^_1_ = 5. 51, *P* = 0.001 and χ^2^_1_ = 182.47, *P*<0.0001, respectively; Figure 2).

Prevention success and promotion success–darkness time and number of treat activations, respectively–were each positively associated with activity level. The association was present at the level of the situation (within-subjects; darkness: *z* = 4.27, *P*<0.0001; treat: *z* = 8.19, *P*<0.0001) and as an individual difference (between-subjects; darkness: *z* = 3.25, *P* = 0.001; treat: *z* = 9.08, *P*<0.0001).

We found the expected individual-level (only) positive association between prevention and promotion success (individual: *z* = 2.00, *P* = 0.045; situation: *P* = 0.78), suggesting that, like humans [15], rats who are highly successful in prevention tend to be also highly successful in promotion. Controlling for activity level, however, we found a situational trade-off such that darkness time was inversely related to number of treats activated (situation: *z* = 2.99, *P* = 0.003; individual: *P* = 0.56). In other words, if on a given day we were to compare rats with the same activity level, we would find a trade-off (negative correlation) between the amount of darkness and the number of treats activated.

The probability of eating all the treats activated was high overall, 74%. At both the individual and situational levels, however, promotion success was associated with an increased probability of eating all treats (logit-link, binomial error; individual: *z* = 2.63, *P* = 0.009; situation: *z* = 2.24, *P* = 0.025). Interestingly, in a multiple regression including both activity level and promotion success, individual differences in activity level predicted a marginally *lower* probability of eating all the treats (*z* = 1.70, *P* = 0.09) while individual differences in promotion success remained significantly predictive of a higher probability of eating all the treats (*z* = 2.97, P = 0.003). Thus, in this case, promotion success and activity level show divergent tendencies.

#### Welfare and RFT

**Figure 3:**
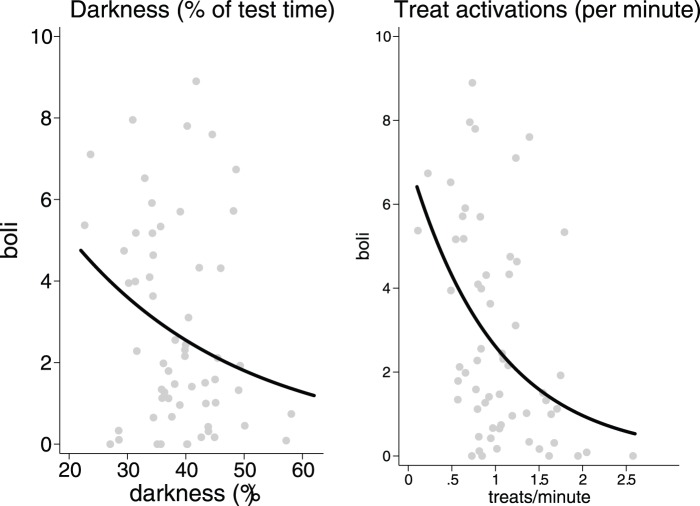
Individual differences and chronic differences in anxiety responses or welfare (black lines are Poisson regression model fit; grey dots are individual means). At the level of the individual (and not the situation), prevention success and promotion success were both significantly related to less fecal boli (*P*<.05 and *P*<.001, respectively).

We used fecal boli production as a potential indicator of poor welfare [35]–[37]. Consistent with acclimatization across test days, fecal boli production progressively decreased (*z* = 4.32, *P*<0.0001) and, as predicted, boli increased following environmental stressors (z = 4.33, P<0.0001). Beyond these situationally induced fluctuations in welfare, rats also showed strong individual differences, which is consistent with chronic differences in fear and anxiety responses and potentially poor welfare (*χ^2^_1_* = 211.19, *P*<0.0001). Moreover, we found that these individual differences in this metric of poor welfare were inversely related to prevention success and promotion success: significantly at the level of the individual (prevention: *z* = 1.99, *P* = 0.047; promotion: *z* = 4.27, *P*<0.0001; Figure 3) and only marginally at the level of the situation (prevention: *z* = 1.77, *P* = 0.076; promotion: *z* = 1.64, *P* = 0.10). Despite the positive association between promotion success and prevention success, multiple regression indicated that both effects were simultaneously true: greater prevention success and greater promotion success were inversely associated with indications of poor welfare (darkness time: *z* = 1.92, *P* = 0.055; treat: z = 3.39, *P* = 0.001).

### Testing Phase 2: Effect of NNO

**Figure 4:**
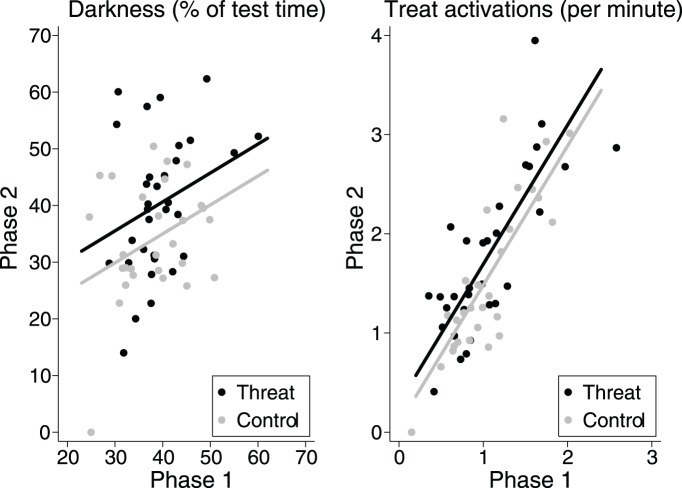
Effect of threatening novel object (lines are average trend by group; dots are individual means). Manageable threats lead to a significant increase in prevention behavior (*P*<.05) and a non-significant increase in promotion behavior.

Two months after Phase 1, rats continued to show significant stability in prevention success and promotion success motivation (prevention *r* = 0.33, *P* = 0.01; promotion *r* = 0.82, *P*<0.0001; Figure 4). Before exposure to the NNO, the two groups were statistically indistinguishable in prevention and promotion behavior (*P*’s >.3). As predicted, being exposed to the manageable threat (the NNO) caused a significant increase in prevention (*t*_58_ = 2.16, *P* = 0.035; Figure 4) but no significant increase in promotion (*P* = .31). Nonetheless, there was a positive correlation between change in prevention success and change in promotion success such that animals demonstrating the greatest increase in darkness time were likely to have greater increases in treat activations as well (*r* = .35, *P* = .006).

Approximately half the animals receiving the NNO (17 of 32), could be classified as persistently or rigidly low prevention animals in that they had low prevention success in Phase 1 and showed low or negative change in prevention in Phase 2. The remaining animals receiving the NNO (15 of 32) either began with high prevention success in Phase 1 and/or showed signs of enhanced prevention in Phase 2. The rigid-low prevention rats showed signs of more stress (more boli) in response to the NNO treatment than the high/plastic prevention rats (Poisson regression: *z* = 2.76, *P* = 0.006).

## Discussion

Extending previous research [20], [21], we found stable individual differences in the safety-maintenance (prevention) and gain-maximization (promotion) behaviors of rats. Operationalized as darkness time (safety for nocturnal animals) and palatable food treat activations, respectively, these individual differences were stable over a relatively short testing period (Phase 1, two weeks) and persisted for at least two months (Phase 2). Previous research in rats has shown stability over a similarly short testing period [21], but the present research is the first demonstration of long-term stability (over two months). Furthermore, over the three test days in Phase 1, we found increases in both the amount of darkness maintained and treats activated, indicating that both outcomes were positively reinforcing for these animals. The complimentary decrease in time spent in the nontreat and light arms strengthens this interpretation. Similar to what is found in humans [28], we found the predicted positive association between individual differences in prevention success and promotion success and an indicator of welfare. Finally, in Phase 2, we demonstrated that exposure to manageable threats (NNOs) increased prevention motivation.

### RFT: Two Types of Approach Motivation

Consistent with the hypothesis that prevention and promotion both reflect approach-type motivations and not avoidance-type motivations, we found that activity was positively associated with both the amount of darkness maintained (prevention success) and the number of treats activated (promotion success). Though these correlations occurred at the situational-level, which alone might have indicated that total darkness and treats were simply the result of high activity level (or vice versa), they were also significant at the individual-level. The presence of an individual-level relationship is important to researchers interested in animal personality or behavioral syndromes because it suggests a more fundamental phenotypic or genetic association [18], [42]. Moreover, the positive individual-level association between activity and prevention motivation distinguishes it from previously described individual differences in avoidance-related phenomenon, such as shyness [14], harm-avoidance [24], or fear [43], which would be negatively related to activity in a novel environment.

Moreover, we found an individual-level positive association between prevention success and promotion success. These patterns may reflect a success-begets-success phenomenon such that success in one domain, e.g. prevention, can enhance an individual’s success in another domain, e.g. promotion. In humans, there is evidence of a modest but significant positive correlation between having a self-reported history of being effective in promotion and being effective in prevention [15], [19]. Similarly, comparing behavior in Phase 1 and Phase 2, we also found a positive correlation between increases in prevention success and increases in promotion success from Phase 1 to Phase 2. However, the relationship between prevention and promotion motivation is not a simple one. In the present experimental paradigm, for example, there was also a spatial-temporal trade-off between focusing on darkness maintenance and treat activations at any particular point in time: a rat could not be in two places at once and thus on a fine-grain scale must choose between prevention success and promotion success. As would be typical in many real world scenarios–for example, foraging vs. hiding in the presence of predators–we find the expected situational trade-off between focusing on attaining gains (treats) and maintaining safety (darkness), but only when holding activity level constant, which, at high levels, can facilitate the pursuit of both goals.

Research has shown that in nonhuman animals, prevention motivation can be distinguished from other individual differences, such as shyness [20], [21]. In an initial step towards discriminating promotion motivation from other personality constructs such as activity level [3], [44] and novelty-seeking [24], we show that greater promotion motivation and not activity level predicted a higher probability of finishing all activated treats. Though rats typically ate the treats they activated, they only ate them all 74% of the time. Various reasons may contribute to why a rat would not finish all activated treats, including but not limited to: satiation, lack of interest, error (the treat rolled out of sight), fear, or distraction (the rat was attending to some other stimulus). Despite the fact the promotion animals activated more treats and thus had more opportunities to experience one of these interferences, we found high promotion predicted a *lower* probability of leaving one behind. This result is an intriguing and potentially counter-intuitive finding because if treat activations were random or simply the result of being an active or novelty-seeking individual, one would expect more treat activations to be associated with a higher probability of leaving one or two behind. Indeed, we found that higher activity was associated with a marginally greater probability of leaving some behind, but promotion significantly predicted the opposite pattern. This finding is consistent with RFT’s proposal that individuals in a promotion focus are motivated to maximize gain rather than just being more active [14]. More research is needed, however, to replicate and extend the distinct nature of promotion motivation from general approach motivation in nonhuman animals.

### Theoretically Driven Models of Animal Personality

With the exponential growth in research on animal personality, there is increased need for theoretical approaches to the study of individual differences in animal behavior [1], [8], [9]. Of particular interest are models that can account for both within-individual and between-individual variation in behavior–behaviors that occur as a result of states as well as traits [9]. Prevention and promotion motivations may be useful in this regard as they can, according to theory and empirical evidence, arise from either states or traits [13], [14]. In other words, the current promotion or prevention motivation of an individual may be the result of a situational pressure or a chronic tendency (personality). Regardless of the source of the motivation–situation or individual–RFT makes the same predictions regarding how prevention versus promotion motivation influences cognition, emotion, and behavior [14], [45].

The transition from a state to a trait has been hypothesized to involve a positive feedback mechanism [33], [34]. A positive-feedback loop can amplify relatively small state differences into large and stable individual differences (personalities). For example, if an animal happens to be successful foraging in a risky patch of land, it will gain resources (e.g., information) and energy that will then increase the probability that it will be able to take future risks. Over time, this feedback loop can stabilize behavior such that the initial risky state becomes a trait or personality [33], [34]. In the present research program, we sought evidence of such a mechanism operating for prevention motivation. Previously, we found that animals with elevated prevention motivation spend more time in close proximity to threatening NNOs [21]. This observation represents the first stage in a potential positive-feedback-loop: a motivation drives an animal to seek out certain environmental conditions. The second stage that would complete the loop would be that these environmental conditions maintain or amplify the initial motivation. To test this hypothesis with regard to RFT, we exposed approximately half the rats to several NNOs between Phase 1 and Phase 2. As predicted, we found evidence consistent with a positive-feedback mechanism: repeated close proximity to threatening NNOs enhanced subsequent prevention motivation (increased in darkness time achieved). Thus, our results indicate that a positive-feedback-loop may contribute to the maintenance of stable prevention motivation.

Future research could investigate positive-feedback-loops in promotion motivation–e.g. do environments that afford gain opportunities enhance promotion motivation? Furthermore, similar patterns involving social niche specialization may operate as well [46]. It is possible that group living may require individuals to adopt vigilant versus eager roles, for example, roles that entail colony defense versus foraging. If so, according to RFT [14], [47], those roles would be sought out by individuals with a prevention versus promotion motivational state, respectively, and may, in turn, sustain or strengthen the individual’s initial motivation. Thus, the combination of behavioral ecology models and RFT can generate testable hypotheses regarding the development of personality in animals.

### RFT and Welfare

Like previous research [35]–[38], we found that rodent fecal boli production varied in response to environmental stress. We also report an inverse relationship between chronic boli production and both darkness maintenance *and* treat attainments, consistent with our hypothesis that current promotion and prevention successes would correlate with signs of better welfare [31]. Nonetheless, stress bears a complicated relationship to welfare and several lines of research have suggested that acutely stressful events are sought out by some individuals [48], [49] and may even be beneficial to welfare [21], [31], [50]. Our hypotheses and results, however, regard chronic differences in stress, which are more likely to reflect poor welfare or the experience of consistently failing to be effective at meeting one’s motivational needs [25]–[27], [31]. In support of this interpretation, we do not find strong associations between promotion and prevention success and acute (situational) signs of stress, but instead find the associations to be strongest at the level of the individual. In other words, we find that some rats show signs of being chronically stressed and that these are the same individuals who are consistently poor at achieving promotion and prevention successes in the maze.

Though the motivational model of welfare has been studied extensively with species-level motivations [25]–[27], [32], it has received relatively little attention at the level of the individual, though there are some exceptions to this pattern [51], [52]. In combination with RFT, we adopt this model of welfare to propose a framework for understanding certain individual differences in animal welfare. Research in humans has shown that a history of being successful in achieving promotion and prevention goals leads an individual to adopt more effective promotion and prevention behaviors in the present [15]. Extending this mechanism to animal welfare, we hypothesized and found evidence consistent with the notion that a history of promotion and prevention successes would lead to greater current promotion and prevention success and greater current signs of good welfare. Importantly, however, RFT does not predict that all individuals will experience promotion and prevention successes to the same extent. Rather, for example, an individual with high promotion motivation will experience a treat or a gain as more of a success than an individual with low promotion motivation. Similarly, an individual with high (vs. low) prevention motivation will experience darkness or safety as more of a success. Understanding how specific environments fit with specific motivational profiles may be an important line of future research in animal welfare science [7], [47].

RFT and the results of the present research may also suggest potential screening and training applications for wildlife reintroduction programs. After experiencing the safety of captivity, animals are at risk for losing the vigilance behaviors that can be life saving in the wild [10], [53]. Our results, along with previous research and RFT, could indicate the potential utility of screening animals for prevention motivation prior to release. Future studies could test whether individuals with strong prevention motivation in captivity are more likely to survive the dangers of relocation and reintroduction. In addition, the results in Phase 2 may suggest a method by which wildlife managers could encourage lower prevention animals to become higher prevention animals. The repeated NNOs served a situational pressure to adopt a prevention motivation (defense of the home-cage) and led to a relatively persistent (one week after treatment) enhancement in prevention motivation. However, these are preliminary results and not all animals responded to the NNOs with increased prevention motivation; more research is necessary to determine the efficacy of this approach. Indeed, the rigidly low prevention animals were the same individuals showing the greatest signs of stress in response to treatment.

Recent work on cognitive bias has also examined how exposure to aversive elements in the environment can shift behavioral responses [54]. Cognitive bias refers to the tendency of an individual to respond to environmental ambiguity regarding potential reward vs. potential punishment in a consistently optimistic (anticipating reward) vs. pessimistic (anticipating punishment) manner. The majority of cognitive bias studies find that exposure to poor quality environments leads to more pessimistic responses, reflecting poor welfare [54]–[56]. Unlike cognitive bias studies, however, we did not design our environmental manipulation to be ultimately negative in that we intended the NNOs to introduce a *manageable* aversive element, one that could eventually be contained and eliminated. Nevertheless, it is possible and even likely that a subset of the individuals in our study were unable to cope effectively with the NNOs. Cognitive bias research and RFT predicts that these individuals would have worse welfare than individuals who were able to cope effectively with the NNOs. Though we have no measure of how well individuals coped with the NNOs, we did observe that the rigidly low prevention individuals showed signs of greater stress than the more plastic individuals, which is consistent with these predictions insofar as plasticity is a component of the ability to cope with environmental pressures.

### Limitations

Though RFT was developed in humans, the present research examines patterns of behavior in a genetically similar line of laboratory-housed animals. There are indications that similar differences exist in non-laboratory, nonhuman species [20], yet care is always required when considering the generalizability of laboratory research. Because selection pressure is severely relaxed or altered in captivity, variability in behavior may not match or even correspond to that found in the field. Of course, it is also possible that the individual differences we found in promotion and prevention will prove to be stronger in the field [57]. Indeed, it is noteworthy that despite high genetic and environmental similarity, stable individual differences of this type emerge in the laboratory. Alternatively, it may be the case that laboratory conditions are precisely what allows these differences to arise; in the wild, the various ecological pressures could serve to smooth out individual variation of this type. Determining how promotion and prevention motivations operate in non-captive populations will be a necessary step in understanding its role in animal behavior.

### Conclusion

In sum, we find evidence that RFT may integrate well with theories in behavioral ecology, behavioral biology, and animal welfare science to provide a deeper understanding of animal personality and welfare. The extensive research in humans combined with the promising preliminary results in other animals indicates that RFT may address fundamentally important differences in motivation. In the present research, we find that RFT (a) may provide unique predictions for behavior, (b) could contribute to the current interest in delineating the causes of personality, and (c) may be a generative model for understanding patterns of animal welfare. Moreover, these results point to several new lines of research investigating the role of RFT in animal behavior more broadly.
